# Dextromethorphan Suppresses Lipopolysaccharide-Induced Epigenetic Histone Regulation in the Tumor Necrosis Factor-*α* Expression in Primary Rat Microglia

**DOI:** 10.1155/2020/9694012

**Published:** 2020-12-05

**Authors:** Yung-Ning Yang, Yu-Chen S. H. Yang, Pei-Ling Wu, Chun-Hwa Yang, Kuang-Che Kuo, San-Nan Yang

**Affiliations:** ^1^Department of Pediatrics, E-DA Hospital, Kaohsiung, Taiwan; ^2^School of Medicine, College of Medicine, I-Shou University, Kaohsiung, Taiwan; ^3^Joint Biobank, Office of Human Research, Taipei Medical University, Taipei, Taiwan; ^4^Graduate Institute of Medicine, College of Medicine, Kaohsiung Medical University, Kaohsiung, Taiwan; ^5^Department of Pediatrics, Kaohsiung Chang Gung Memorial Hospital and Chang Gung University College of Medicine, Kaohsiung, Taiwan

## Abstract

The activation of microglial cells plays an important role in the cascade of events leading to inflammation-mediated neurodegenerative disorders. Precision therapeutics require that adjunctively feasible drugs be found to prevent microglial cell activation and prevent inflammation-mediated neuronal injury. Dextromethorphan (DM) has been reported to possess neuroprotective effects in lipopolysaccharide- (LPS-) stimulated animals; however, it remains unclear whether epigenetic regulatory mechanisms in microglial cells are involved in such DM-mediated neuroprotective effects. In this study, DM simultaneously suppressed LPS-induced activation of tumor necrosis factor- (TNF-) *α* expression and subsequent caspase-3 signaling in primary microglial cells associated with notable morphological changes. Furthermore, therapeutic action sites of DM involved differential enhanced trimethylation of H3K4 modifications in the promoter region of *tnf*-*α* gene locus in primary microglial cells. In summary, DM may exert neuroprotective and anti-inflammatory effects through differential epigenetic histone modifications of TNF-*α* expression in microglial cells and might therefore raise the possibility of providing an adjunctively beneficial role for a tentative therapeutic strategy in neurodegenerative diseases resulting from inflammation.

## 1. Introduction

The microglial cell serves as an important initiator of pathophysiological cascades leading to neuroinflammation through proinflammatory mediators within the central nervous system (CNS) [1]. Microglial cells can be activated by cytokines and/or chemokines and thereby release proinflammatory factors including interleukin-1*β* (IL-1*β*), interleukin-6 (IL-6), nitric oxide synthase (iNOS), tumor necrosis factor-*α* (TNF-*α*), and cyclooxygenase-2 (COX-2) [2, 3]. Also, the microglial activation is reported as participating in several neurodegenerative diseases, such as multiple sclerosis, Parkinson's disease, and Alzheimer's disease [4, 5].

Lipopolysaccharides (LPS) is a membrane structural constituent of gram-negative bacteria and a well-known endotoxin and lipoglycan. Bacterial LPS can trigger a cascade of biochemical events of the innate immune system and enhance an expression of microglia-derived proinflammatory factors, such as TNF-*α*, a crucial mediator of inflammation-mediated neurodegeneration [6]. The control of TNF-*α* production is recognized via genetic variations, signaling networks, and epigenetic regulation of TNF-*α* expression [7]. It has long been known that the epigenetic regulatory mechanisms include DNA methylation, histone modification, and noncoding RNAs. The expression of TNF-*α* messenger RNA and protein was reported by epigenetic modifications of the TNF-*α* promoter gene through DNA methylation or chromatin remodeling [8]. Trimethylation of H3K4 contributes significantly in the initial recruitment of a methyltransferase complex that associates with the P65 protein of the NF-*κ*B family and mediates dimethylation to trimethylation conversion of histone H3 at K4 [9]. The NF-*κ*B had reported regulated its downstream gene transcription such as TNF-*α* [10]. Additionally, our previous reported that bacterial LPS could enhance trimethylation of histone 3 at lysine 4 (H3K4me3) in the TNF-*α* promoter gene locus associated with neuronal apoptotic damage in the neonatal rat brain [8]. Furthermore, such a bacterial LPS-induced increase in TNF-*α* expression caused an activity-dependent neuronal apoptosis in the neonatal cerebral cortex [11, 12]. Thus, feasible therapeutic strategies need to be found to prevent microglial cell activation and avoid inflammation-mediated neuronal injury.

Dextromethorphan (DM), an antitussive agent, has been widely used in clinical patients for more than half a century. DM acts as a low-affinity noncompetitive *N*-methyl-D-aspartate (NMDA) receptor antagonist [13, 14], sigma-1 receptor agonist [15], and *α*3*β*4-nicotinic receptor antagonist [14]. We previously revealed that administrations of DM could reduce the reward effects and drug-seeking effects due to morphine [14, 16] or methamphetamine [14, 17] abuse in an animal model. In addition, DM could also exert neuroprotective effects through decreases in TNF-*α*, IL-1*β*, and IL-6 protein expressions following various CNS insult paradigms, including seizure, ischemic, and/or traumatic brain injury [14, 18]. DM could inhibit LPS-induced microglial activation and block methamphetamine-induced microglial activation *in vivo* [19], although it remains unclear whether DM can alleviate the enhanced expression of H3K4me3 in the TNF-*α* promoter gene locus in LPS-stimulated primary microglial cells; accordingly, this study was designed to examine whether DM exerts anti-inflammatory effects by attenuating LPS-activated epigenetic histone modifications in the TNF-*α* expression in primary enriched microglial cells, thereby providing a potentially beneficial role for adjunctive therapeutics in neurodegenerative diseases resulting from inflammation through microglial cell activation.

## 2. Materials and Methods

### 2.1. Primary Microglial Cell Culture

Primary enriched microglial cells were prepared from 1-day-old Sprague Dawley (SD) rat [20]. In detail, the whole brains of 1-day-old pups were dissociated in a 0.25% Trypsin-EDTA solution for 10 minutes (37°C). The digested microglial cells (1 × 10^7^) were filtered through a 100-*μ*m-pore mesh, pelleted, and seeded in 75 cm^2^ poly-D-lysine-precoated flasks in 20 mL of a Dulbecco's modified Eagle's medium/F12 mixture (1 : 1) containing 10% heat-inactivated fetal bovine serum, 2 mM, L-glutamine; 1 mM, sodium pyruvate; 100 *μ*M, nonessential amino acids; 50 units/mL, penicillin/streptomycin. Cells were cultivated in flasks, and medium was refilled 3~4 days after original seeding. Enriched microglial cells were detached by trembling at 150 rpm for 5 h after a confluent monolayer had formed (10~14 days) and were planted in 12-well plates at a density of 10^5^ cells/cm^2^. After introduction to serum-free medium for 24 h, enriched microglial cells were exposed to a single concentration of LPS (0.2 *μ*g/mL) and/or DM (1 *μ*M) for 24 h. The purity of the microglia was >98% as assessed by OX-42 immunostaining (a specific microglial cell marker). Upon reaching confluence (day 19-20), microglia were shaken off and plated (10^5^/well) into 24-well culture plates. Twenty-four hours later, cells were used for drug treatments. LPS (Escherichia coli 0111:B4) were purchased from Sigma Chemicals Company (St. Louis, MO, USA).

### 2.2. Immunocytochemistry

At the end of the trial protocol period, cultures grown in 24-well plates were washed once with phosphate-buffered saline (PBS) and fixed for 20 min at room temperature in paraformaldehyde (3.7% in PBS). After washing with PBS, cultures were treated with hydrogen peroxide (1% for 10 min). Cultures were washed with PBS and then incubated for 40 min with blocking solution (PBS containing 0.4% Triton X-100, 1% bovine serum albumin, and 4% normal horse serum). Microglial cells were then hatched overnight (4°C) with the primary antibody OX-42 (Catalog No.554859. BD Biosciences, San Jose, CA, USA) diluted in blocking solution. Subsequently, enriched microglial cells were washed by PBS (10 min) and then hatched for 2 h with PBS containing 0.3% Triton X-100 and an appropriate biotinylated secondary antibody (Catalog No.BA-2001. Vector Laboratories, Burlingame, CA, USA). Enriched microglial cells were incubated with the Vectastain ABC reagent (40 min) and visualized with 3,3′-diaminobenzidine (DAB) and urea-hydrogen peroxide tablets, according to the manufacturer's suggestion (Sigma Chemical, USA). Immunocytochemistry imaging was determined by a Nikon diaphot-inverted microscope system.

### 2.3. Immunoblotting

Enriched microglial cells were flushed in cell lysis buffer (0.2% Triton X-100,10 mM Tris at pH 7.4, 2 mM EDTA, 150 mM NaCl, 1 mM phenylmethanesulfonylfluoride, and 1x protease inhibitor mixture). The protein concentration was analyzed using a bicinchoninic acid assay (Thermo Scientific, USA). Cell lysates were separated on sodium dodecyl sulfate-polyacrylamide gel electrophoresis (SDS-PAGE) and then transferred to nitrocellulose membranes. They were probed with an antibody against TNF-*α* (SC-1351, Santa Cruz Biotechnology, USA) and cleaved caspase-3 (#9662, Cell Signaling, USA). Our secondary antibodies were either rabbit anti-mouse IgG or goat anti-rabbit IgG (1: 3000), depending on the primary antibody. For quantification of immunoblot signals, the band intensity was assessed using the Kodak Digital Science 1D program (Rochester, USA).

### 2.4. Chromatin Immunoprecipitation (ChIP) Assay

Chromatin immunoprecipitation assay was achieved as previously reported [8]. Briefly, 5 × 10^5^ cells were incubated for 10 minutes at room temperature with 1% formaldehyde, followed by sonication of DNAs and the immunoprecipitation of chromatin overnight with the selective antibody for trimethylated H3K4 (2 *μ*g for 25 *μ*g of chromatin; anti-histone H3K4me3 antibody, ChIP Grade; ab8580; Abcam Co.) and then purification with the ChIP kit (ChIP kit number 17-295; Upstate Biotechnology, Lake Placid, NY, USA). The primers were deliberated by evaluating the proximal promoter and intronic enhancer regions of the TNF-*α* gene, encompassing the following subregions relative to the transcription start site: TNF-*α* (1) (-2686 to -2667); Forward, 5-CCCTAGTCCTCCTGGGATGT-3 and Reverse, 5-GCCTGCTGCAACAGAGAGA-3; TNF-*α* (2) (-2202 to -2183); Forward, 5-CGTCTCACTATGCCTGGGTCT-3 and Reverse, 5-AAGCAAAGCACTTCTACCAAAT-3; TNF-*α* (3) (-1672 to -1653); Forward, 5-AAACTCAGACCAGGCTGCAT-3 and Reverse, 5-CAGGTCATCTCTTGACGTGGT-3; TNF-*α* (4) (-502 to -480); Forward, 5-GAGTTCTGCATGTATTGGATAGG-3 and Reverse, 5-TGCTACCAAGCCTAAAGACC-3; TNF-*α* (5) (-230 to -213); Forward, 5-GGTTCAGTTCCCAGCACCTA-3 and Reverse, 5-ATGGGCATATCTGCACAGCA-3. The PCRs were achieved on the ABI Gene Amp^ⓐ^ PCR System (Applied Biosystems). The amount of immunoprecipitated DNA is calculated compared with the total input DNA.

### 2.5. Statistical Analysis

All data in this study are shown as the mean ± standard error of mean (SEM). Statistical differences were assessed by a commercial software (SigmaState 3.5) for the analysis of variance (ANOVA) with repeated measurements followed by Bonferroni's *t*-test for post hoc multiple comparisons. The level for a statistical significance (*p* < 0.05) was used to all tests.

## 3. Results

### 3.1. Cell Morphology in Primary Microglial Cell Culture

**Figure 1:**
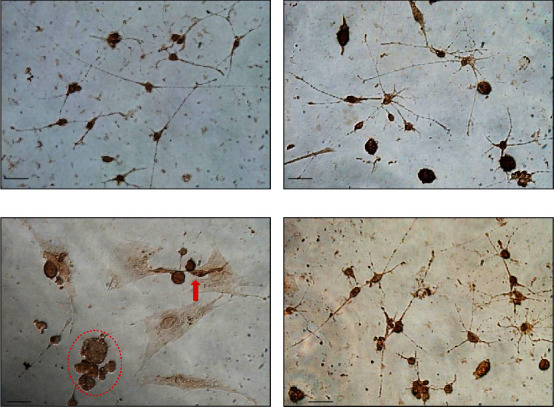
Immunohistochemical morphology evaluated in primary microglial cells. Experiments were designed as four experimental groups as follows: vehicle-control (inset (a)), dextromethorphan (DM) alone (inset (b)), lipopolysaccharide (LPS) alone (inset (c)), and DM+LPS (inset (d)). Scale bar equals 50 *μ*m.

The validation of microglia cell purification was tested >98%, as assessed by OX-42 immunostaining (a specific microglial cell marker). The immunohistochemical (IHC) morphology was evaluated in primary microglial cells. Briefly, experiments were designed as four groups as follows: vehicle-control, DM alone (1 *μ*M for 24 h), LPS alone (0.2 *μ*g/mL for 24 h), and a 0.5-h period of pretreatment with DM (1 *μ*M) followed by a 24-h period of LPS exposure (0.2 *μ*g/mL). Primary microglial cells were then fixed for biochemical and immune-staining experiments. As shown in Figure 1, immunocytochemical staining (OX-42, a specific microglial cell marker) predominantly revealed microglial cells in primary cultures. In a relatively resting state (vehicle-control, Figure 1(a)), most microglial cells appeared to have a bipolar morphology with long and slender processes. In comparison, when exposed to LPS (Figure 1(c)), most microglial cells appeared to be activated, with hypertrophic cell bodies with many vacuoles in the cytoplasm, and thickened and shrunken processes [19]. As illustrated in Figure 1(d), pretreatment with DM significantly prevented such LPS-induced morphological changes in primary enriched microglial cells as shown in Figure 1(c), whereas DM alone had no significant effect on morphological appearances (Figure 1(b)).

### 3.2. DM Reduced the Enhancement of LPS-Activated TNF-*α* Expression

**Figure 2:**
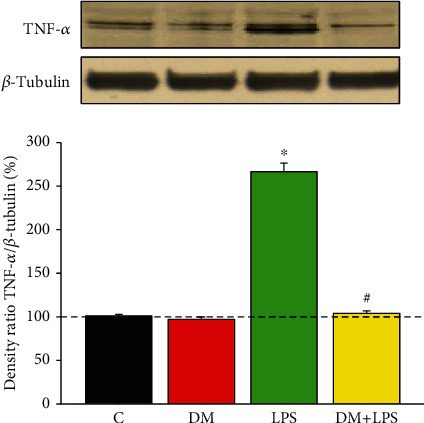
Dextromethorphan alleviated the increase in LPS-induced TNF-*α* expression in primary microglial cells. Lysates were probed by immunoblotting with antibodies against TNF-*α* and *β*-tubulin (which served as an internal loading control). The levels of TNF-*α* expression were shown as the ratio of the TNF-*α*/*β*-tubulin signal intensity for each experiment. Each group was derived from ten independent experiments. The experimental groups were as follows: (C) control, (DM) DM alone, (LPS) LPS alone, and (DM+LPS) DM plus LPS. ^∗^*p* < 0.05, compared to the control group. ^#^*p* < 0.05, compared to the LPS-alone group.

The levels of TNF-*α* expression were detected in primary enriched microglial cells. As shown in Figure 2 (*n* = 10 independent experiments for each group), in the LPS-alone group, LPS significantly enhanced TNF-*α* expression, compared to the control group (*n* = 10, *p* < 0.05). In the DM-alone group, DM by itself exerted no notable effect on TNF-*α* expression. In contrast, in the DM+LPS group, DM treatment significantly attenuated LPS-induced enhancement in TNF-*α* expression compared to the LPS-alone group (*n* = 10, *p* < 0.05; Figure 2).

### 3.3. DM Reduced LPS-Activated Histone Modifications at the TNF-*α* Promoter Gene

**Figure 3:**
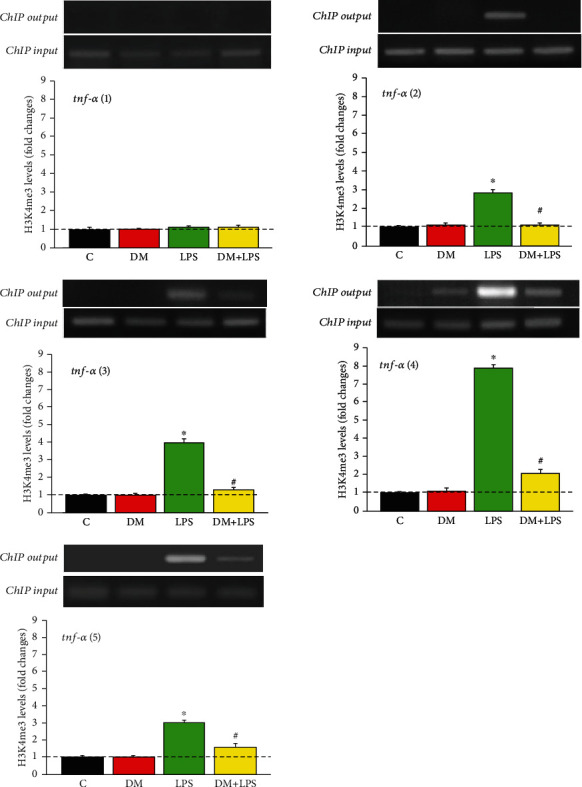
Dextromethorphan reduced the LPS-activated enhancement of trimethylated H3K4 expression in TNF*-α* promoter gene. The TNF-*α* promoter gene locus included the following subregions relative to the transcription start site: five subregions *tnf*-*α* (1~5). The experimental groups were as follows: (C) control, (DM) DM alone, (LPS) LPS alone, and (DM+LPS) DM plus LPS. The levels of H3K4me3 output were normalized to loading input which served as the internal loading control. ^∗^*p* < 0.05 compared to the control group. ^#^*p* < 0.05 compared to the LPS-alone group.

Epigenetic modifications regulate the expression of TNF-*α* promoter gene in response to acute stimulation such as by LPS [21, 22]. Previous study reported that acetylated H3 and H4 activated transcription effects in the TNF*-α* gene locus of monocytes and macrophages [21]. To examine whether the TNF-*α* gene locus underwent histone modifications in microglial cells as the result of LPS treatment (0.2 *μ*g/mL), we performed ChIP analyses of primary enriched microglial cells treated with LPS, using PCR primers corresponding to five subregions (TNF-*α* 1~5) in the TNF-*α* promoter gene locus. In Figure 3 (*n* = 10 independent experiments for each group), differentially upregulated levels of trimethylated H3K4 at the promoter subregions of TNF-*α* (2~5) were noted in the LPS-alone group (*n* = 10, *p* < 0.05). However, in the DM+LPS group, a significant decrease in H3K4me3 expression in the TNF-*α* gene locus was observed, indicating that DM was likely to alleviate the LPS-activated microglial activation via differential epigenetic regulation in the TNF-*α* promoter, at least in part.

### 3.4. DM Reduced the Enhancement of LPS-Activated Caspase-3 Expression

**Figure 4:**
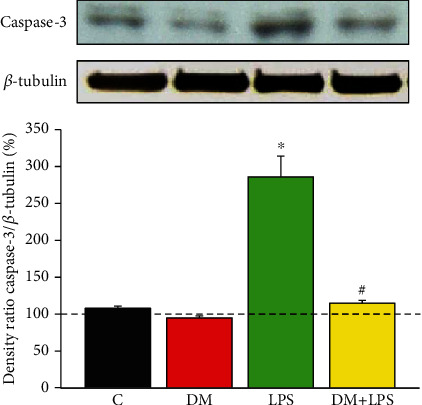
Dextromethorphan reduced LPS-activated enhancement in caspase-3 expression in primary microglial cells. Samples were probed by immunoblotting with antibodies against cleaved caspase-3 and *β*-tubulin (which served as the internal loading control) were indicated by the ratio of the caspase-3/*β*-tubulin signal intensity in each group. Each group was derived from at least 10 independent experiments. The experimental groups were as follows: (C) control, (DM) DM alone, (LPS) LPS alone, and (DM+LPS) DM plus LPS. ^∗^*p* < 0.05, compared to the control group. ^#^*p* < 0.05, compared to the LPS-alone group.

We simultaneously determined whether DM also exerted beneficial effects on LPS-activated caspase-3 expression (an important apoptosis signaling pathway) in primary enriched microglial cells. As shown in Figure 4 (*n* = 10 independent experiments for each group), the levels of cleaved caspase-3 were significantly enhanced in the LPS-alone group (*n* = 10, *p* < 0.05), while DM reduced the LPS-activated enhancement in cleaved caspase-3 in the DM+LPS group (*n* = 10, *p* < 0.05).

## 4. Discussion

The present findings suggest that DM treatment effectively attenuated LPS-activated alterations in the proinflammatory cytokine TNF-*α* apoptotic signal caspase-3, and morphological characteristics in primary microglial cells. Furthermore, such beneficial effects of DM therapy described above were likely, at least in part, through epigenetic H3K4me3 modifications in the TNF-*α* expression.

The activation of microglial cells plays an important role in neural parenchymal defense counter to diseases, such as inflammation, trauma, ischemia, brain tumor, and neurodegeneration [23]. Activated microglial cells may secrete various proinflammatory cytokines and neurotoxic factors that are theorized to induce neurodegeneration [24]. Therefore, it is important to identify novel therapeutic strategies in which the decreased microglial overactivation with a downstream inhibition of proinflammatory cytokines and enzymes releases may be a useful therapeutic tactic for the effective treatment of neurodegenerative disorders. Indeed, previous studies suggested that DM, an antagonist of the *N*-methyl-D-aspartate (NMDA) receptor complex, can exert neuroprotective effects [25–28]. The mechanisms responsible for the neuroprotective activity of DM were hypothesized to occur primarily through an antagonistic effect on the NMDA receptor [29]. However, explanations of the numerous observed beneficial effects are difficult because identification of high- and low-affinity binding sites for DM in the CNS is problematic [30, 31]. Using primary microglial cells, this study revealed a possibility that the neuroprotective effects of DM in LPS-induced inflammation, at least in part, involved the downregulation of epigenetic histone mechanisms in proinflammatory cytokines in microglial cells, thereby suppressing the production of neurotoxic factors, such as TNF-*α* with subsequent caspase 3.

Activated caspase-3 is crucial signaling for cell apoptosis and CNS inflammation [32]. A stimulation of microglial cells through LPS exposure was demonstrated to be nontoxic to neighboring neurons when caspase-3 was inhibited. Furthermore, initiation of nuclear factor-*κ*B (NF-*κ*B) by caspase-3 is critical in such LPS-induced inflammation [10, 33, 34]. Thus, in the present study, the effects of DM on the caspase-3 expression were determined in microglial cells. Results demonstrated that DM treatment following LPS stimulation markedly inhibited the expression of caspase-3, proposing that such anti-inflammatory effects of DM may be a possible consequence of an inhibition of the NF-*κ*B signaling pathway. This study revealed LPS-activated enhancement of TNF-*α* and caspase-3 production in primary microglial cells, consistent with previous results [8, 22]. Nevertheless, there are few studies to address whether DM treatment for such LPS-stimulated TNF-*α* expression involves epigenetic histone regulation (H3K4me3) in microglial cells of the CNS.

Studies revealed that epigenetic histone modifications regulate gene expression through specific sites over promoter in response to various environmental stimuli [21]. However, most studies have addressed the global changes of epigenetic modification rather than assuming an approach investigating a specific gene. Previously, higher possibilities of the DNA methylation located in specific loci of the TNF-*α* promoter gene as detected from human gingiva with periodontitis [7, 35]. In addition, LPS exposure could activate different but focused regions of histone modification including the methylation in the promoter region of the TNF-*α* gene [21]. Here, this study further disclosed the differential enhancement of H3K4me3 modifications in the TNF-*α* promoter gene locus in microglial cells could be attenuated by DM treatment, and this enhancement over the different promoter locus in TNF-*α* promoter gene following the LPS stimulation might imply that respective transcriptional factors can be recruited into transcription and become affected by various environmental insults. However, it is still worth noting as a research limitation for this study where we only investigated several aspects of DM on epigenetic H3K4me3 modification in LPS-induced TNF-*α* expression, so a conclusion cannot be drawn that DM attenuates activation of microglial cells through epigenetic modifications in TNF-*α* at the transcription level. Thus, continued research is still required to understand which transcription factors are involved and affected by epigenetic histone modifications.

## 5. Conclusions

**Figure 5:**
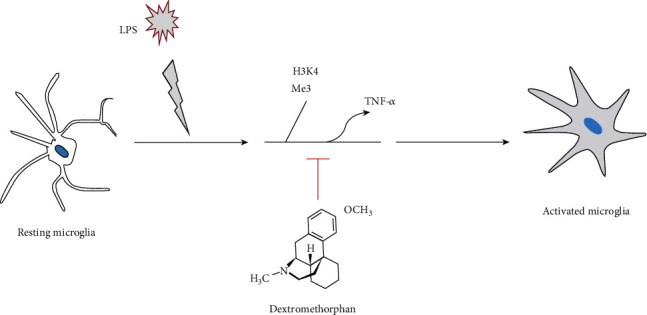
Illustration of the hypothetical scheme for how dextromethorphan attenuates LPS-induced enhancement of TNF-*α*, and hence may be involved in cascades of events leading to an inhibition of microglial cell activation.

This study offers new insights into how DM therapy can recover changes resulting from LPS exposure through reducing inflammation and apoptotic reactions. Even more importantly, the beneficial effects of DM were shown to occur through epigenetic histone regulation (H3K4me3) of TNF-*α* expression (Figure 5). Collectively, this study suggests DM as an adjunctive neuroprotective strategy that might be well-tolerated with fewer side effects on mammalian brains.

## Data Availability

All data used to support the findings of this study are included within the article.

## Abbreviations

DM: Dextromethorphan
CNS: Central nervous system
IL-1*β*: Proinflammatory factors including interleukin- (IL-) 1*β*
TNF-*α*: Tumor necrosis factor- (TNF-) *α*
CXCL10: Interferon gamma-inducible protein-10
ROS: Reactive oxygen species
NO: Nitric oxide
LPS: Lipopolysaccharides
NMDA: *N*-methyl-D-aspartate
H3K4me3: Trimethylation of histone 3 at lysine 4
NF-*κ*B: Nuclear factor-*κ*B
HSP60: Heat shock protein 60.

## Additional Points

Institutional mailing address: 1 Yida Road, Yan-Chao District, Kaohsiung 824, Taiwan.
